# Defect-Engineered Al_2_CO/Al_2_Se_3_ Heterostructure for Enhanced Photocatalytic Water Splitting

**DOI:** 10.1021/acsomega.5c09075

**Published:** 2025-12-23

**Authors:** Iram Shahzadi, Abdul Majid, Bisma Wasim, Mohammad Alkhedher, Ahmed Ahmed Ibrahim, Sajjad Haider, Kamran Alam

**Affiliations:** † Department of Physics, 128417 University of Gujrat, Gujrat 50700, Pakistan; ‡ Mechanical and Industrial Engineering Department, 105947 Abu Dhabi University, Abu Dhabi 59911, United Arab Emirates; § Department of Physics and Astronomy, College of Science, 37850 King Saud University, P.O. Box 2455, Riyadh 11451, Saudi Arabia; ∥ Chemical Engineering Department, College of Engineering, King Saud University, P.O. Box 800, Riyadh 11421, Saudi Arabia; ⊥ Department of Chemical Engineering Materials Environment, 9311 Sapienza University of Rome, Rome 00185, Italy

## Abstract

In this study, we investigate the influence of intrinsic defects on the photocatalytic properties of the Al_2_CO/Al_2_Se_3_ heterostructure using first-principles calculations. The intrinsic defects in the form of oxygen and carbon vacancies in Al_2_CO monolayer and interface Al_2_CO/Al_2_Se_3_ appeared to increase the electronic bandgap; however, aluminum vacancy caused a semiconductor-to-metal transition in the material. The introduction of oxygen vacancies caused charge transfer from Al_2_Se_3_ to Al_2_CO, causing electronic stabilization and revealing the van der Waals interaction in the heterojunction. The band edge alignment of the pristine Al_2_CO monolayer indicated unsuitability for hydrogen evolution, but for the heterojunction, the appearance of oxygen vacancies modified the band diagram and the origin of gap states enabling the heterojunction to trigger water reduction. The modeling of photocatalytic water splitting revealed that the heterostructure containing oxygen vacancies supports hydrogen evolution reaction (HER) and oxygen evolution reaction (OER). The HER is found to be thermodynamically promising on Al and Se sites, indicating Δ*G* of −0.091 eV and −0.144 eV, respectively, whereas the OER presented an overpotential of 1.08 V.

## Graphical abstract

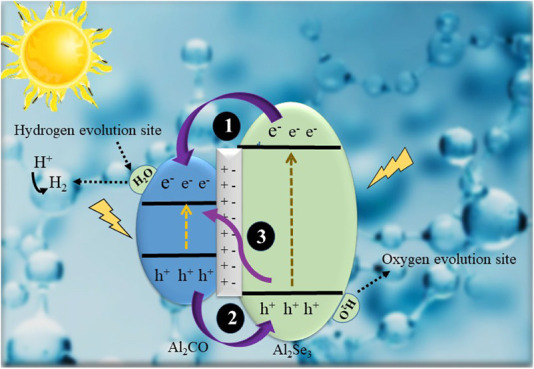

## 1 Introduction

The concerns about environmental degradation and the utilization of conventional energy sources noticeably surface when impending industrialization and future energy demands are taken into account. In the search for renewable energy sources, the production of hydrogen by harvesting solar energy via photocatalysis has attracted great attention. Hydrogen can be produced from many different ways, such as electrolysis, natural gas refining, photobiological water splitting, and photoelectrochemical water splitting.^1^ The environmental consequences and energy efficiency are influenced by the way it is produced. The process of photocatalysis involves a semiconductor-based photocatalyst and photogenerated carriers taking part in the redox reaction of water.^2,3^

The efforts to circumvent the limited absorption of light by photocatalysts involve doping, defect engineering, decoration with metal nanostructures, and the formation of composites.^4^ The challenges related to stability, charge carrier separation, and photocatalytic activity can be addressed via the preparation of composites comprising suitable semiconductors.^5^ Photocorrosion can be avoided via surface modification, composites of different semiconductors, the use of cocatalysts, and postgrowth processing of photocatalysts.

Two-dimensional (2D) materials are getting focal attention because of their diverse and tunable properties when compared to their bulk counterparts.^6^ The 2D materials, in the form of lateral and vertical heterojunctions, are capable of accomplishing properties that are not available via the individual layers. The formation of heterojunctions using different materials leads to the introduction of strain owing to lattice mismatch between the components. Point defects in the form of vacancies, can be exploited in favor of photocatalysis since they can alter the properties of the resulting heterostructure.^7,8^

The defect engineering greatly influences the functionality of electronic devices consisting of the interface and the monolayer. The tailoring of electronic properties driven by defect engineering is capable of increasing the mobility of photogenerated charge carriers and the production of more active sites to enhance the photocatalytic activity on the basis of interactions between defects, adsorbates, and adsorbents. The controlled introduction of point defects has been widely used in the semiconductor industry to modify the electronic properties and supplement the desired electrical conductivity in the materials.^9^ In this regard, the vacancies that prompt surface reactions are of great interest. The vacancies may be anionic, cationic, or both, which are named as multivacancy or the coexistence of anion and cation vacancies. Anion vacancy, consisting of O, S, or halogen vacancy, is useful in catalysts that are metal-based as these may have a low formation energy, which is good for stability.^10^ Cation vacancy is the metal vacancy, and these have the potential to create p-type conductivity, as an upward shift is usually observed in the valence band maximum with no new intermediate state; hence, it favors the hole (h^+^) migration. The intrinsic defects in the form of oxygen vacancies in oxide semiconductors can be produced via controlling the synthesis conditions and ex-situ methods.^11^ The tailoring of the catalytic properties of Sn-doped CeO_2_ nanostructures via oxygen vacancy modulation has been reported. The synergistic effects of metal cations have been shown to modify the concentration and spread of oxygen vacancies in LaCoO_3_.^12^ The presence of vacancies can assist in enhancing the active sites to adsorb water molecules. Studies investigated TiO_2_ samples in pristine as well as with varying oxygen vacancy contents to explore the role of the vacancies in photocatalysis, and an increase in charge transfer rate has been observed.^13^ The enhancement in hydrogen evolution in pure ZnO and Mn/ZnO nanostructures is also attributed to oxygen vacancies.^14^ The vacancy-rich samples of g-C_3_N_4_/ZnS have been reported to exhibit 30 times higher photocatalytic activity with higher absorption of visible light when compared with pristine g-C_3_N_4_.^15^ The absorption rate increases due to the production of gap states, which serve as stepping stones for photon absorption. These also act as trapping centers for photogenerated carriers to increase the carrier’s separation. The exploration of efficient but cost-effective heterojunctions prepared via earth-abundant elements has received special attention recently. Al_2_CO-based heterojunctions have shown excellent photocatalytic activity for overall water splitting under visible light irradiation. This work is dedicated to the exploration of a novel heterostructure Al_2_CO/Al_2_Se_3_ as a photocatalyst with emphasis on the effects of vacancies. The intriguing band alignment characteristics of Al_2_Se_3_, coupled with its minimal lattice mismatch with Al_2_CO, drew our attention to this particular heterostructure. This combination exhibits superior visible-light absorption and efficient charge-carrier separation, making it a more promising candidate than the conventional Al_2_CO/TiO_2_ heterostructure, which typically forms a type-I band alignment that limits photogenerated charge transfer across the interface. In contrast, the Al_2_CO/Al_2_Se_3_ system supports a more favorable type-II (staggered) band configuration, promoting spatial separation of electrons and holes—an essential feature for enhancing photocatalytic and optoelectronic performance.

Furthermore, while Ga_2_O_3_-based van der Waals (vdW) junctions have been widely investigated for similar applications, they often suffer from surface oxidation and poor interfacial coupling, which severely compromise their chemical stability and device reliability. The Al_2_CO/Al_2_Se_3_ interface, however, demonstrates a strong interlayer binding energy of −5.349 eV, indicative of robust interfacial adhesion and excellent structural compatibility. This stability arises from the intrinsic resilience of Al_2_CO, while the inclusion of the chalcogenide Al_2_Se_3_ contributes tunable optical and electronic properties owing to its narrower bandgap and higher light-harvesting capability.^16^ Altogether, the Al_2_CO/Al_2_Se_3_ heterostructure effectively bridges the mechanical and chemical stability of Al_2_CO with the optical tunability and charge transport advantages of Al_2_Se_3_, setting it apart from previously explored III–VI semiconductor interfaces, such as Ga_2_O_3_/MoS_2_. This synergy highlights its potential as a stable, efficient, and optically responsive platform for next-generation photocatalytic and optoelectronic devices.

The comprehensive first-principles calculations are carried out in order to prepare the heterojunction in different stacking configurations, thereby investigating the structural, electronic, and optical properties.

## 2 Methodology

The work reported herein was carried out via first-principles calculations on the Al_2_CO monolayer and Al_2_CO/Al_2_Se_3_ heterojunction in the framework of density functional theory (DFT) implemented within the ADF-BAND package, which employs a linear combination of atomic orbitals (LCAOs).^17^ For large supercell optimization to perform the prescreening of lattice match, the SCC-DFTB3 was used to significantly lower the computational cost. The NEB is the framework of calculation employed within ADF for the determination of transition states and barriers for interfacial charge transfer.^11^ It is well known that the application of hybrid functionals like HSE06 offers an accurate band gap while mitigating the self-interaction errors in the conventional GGA level of theory. Although, their application to large-scale Al_2_CO/Al_2_Se_3_ heterostructures is computationally prohibitive due to the high cost associated with evaluating exact exchange interactions. Given the large supercell required to minimize lattice mismatch and ensure realistic interfacial modeling, full HSE06 calculations have become impractical. However, the application of GGA-PBE has been commonly used, offering a good balance of speed and reliability to predict the electronic properties of catalysts and determine Gibbs free energy, overpotential, and the potential-determining step. The exchange-correlation functional was approximated using the Generalized Gradient Approximation (GGA) developed by Perdew–Burke–Ernzerhof (PBE), supported by Grimme’s D3 dispersion correction (PBE-D3) to account for the long-range van der Waals interactions.^12,13^ The molecular orbitals were expanded in the form of Slater-type basis functions via a triple-ζ polarization (TZP) basis set.^18^ In order to correctly describe the chemical bonding, the calculations were performed using a basis set comprising polarization functions, which ensures the flexibility of the atomic orbitals to form bonds in all directions, thereby improving the accuracy of the electronic structure and geometry of the structures. A vacuum layer of 20 Å was applied along the *z*-direction to eliminate spurious interlayer interactions. The Brillouin zone was sampled using a Monkhorst–Pack *k*-point mesh of 9 × 9 × 1 for monolayers and 7 × 7 × 1 for heterostructure optimizations and DOS calculations. The convergence criteria for energy and step size were 10^–5^ eV and 0.001 Å, respectively. The quality of the *K*-space grid was defined as gamma-only, depending upon the size of the unit cell to sample the Brillouin zone. The plane-wave cutoff energy was set to 500 eV, and all structures were relaxed until the force on each atom was below 0.01 eV/Å. The calculations for Al_2_CO consisting of a vacancy were employed without the frozen core approximation to consider all electrons.

In order to investigate the thermodynamic properties of the materials, the time-dependent behavior is modeled using molecular dynamics (MD) simulations implemented via the DFTB method. The third-order correction is employed via the Self-Consistent Charge (SCC-DFTB) scheme under the DFTB3 level of theory.^15^ The simulation of the system under a constant temperature environment is carried out using a Nosé-Hoover Chain (NHC) thermostat, which maintains the canonical (NVT) ensemble. The damping constant of 5 ps was selected to implement the thermostat, whereas the simulation was performed for 10,000 steps with a time step of 1.0 fs to ensure a complete simulation duration of 20 ps. The comparison of initial and final configurations helped to analyze the structural and energetic properties of the materials.

The calculation of the absorption coefficient was carried out using eq 1.

1 $$\alpha \left(\omega \right)=\frac{\sqrt{2\omega }}{c}{\{{[\varepsilon_{1}^{2}\left(\omega \right)+\varepsilon_{2}^{2}\left(\omega \right)]}^{1/2}-\varepsilon_{1}\left(\omega \right)\}}^{1/2}$$

Whereas *ω* and *c* are the angular frequency and the speed of light, *ε*_1_ and *ε*_2_ are the real and imaginary parts of the dielectric constant, respectively. The charge transfer can be investigated by the charge density difference, as given in eq 2.

2 $$\Delta \rho =\rho_{Al_{2}\mathrm{CO}/\mathrm{ML}}-\rho_{Al_{2}\mathrm{CO}}-\rho_{ML}$$

In order to accurately predict the activation energy barrier (*E*_a_) for quantifying the reaction kinetics under transition state (TS) theory, we performed ab initio calculations on the transition state of the chemical reactions involved. The modeling of the chemical reactions is performed via monitoring the initial state (i.e., reactant) and the final state (i.e., product) to obtain the minimum energy path (MEP). This computational methodology is suitable to provide an understanding of the atomic-scale reaction dynamics related to the role of O-vacancies in catalytic properties and the redox performance of the heterostructure.

## 3 Results and Discussion

The material modification techniques involving defect engineering have been extensively employed to tailor properties in favor of different applications.^19^ Recent studies have provided valuable insights into the behavior and implications of oxygen vacancies in various materials. For instance, a study explored the role of oxygen vacancies in enhancing the catalytic properties of metal oxide surfaces.^20^ Similarly, the influence of oxygen vacancy concentration on the electronic properties of nanostructured semiconductors.^21,22^ These findings align with our observations and underscore the significance of oxygen vacancies in tailoring material properties for a desired specific application.^20,21^ This work was carried out to investigate the effect of intrinsic point defects on the properties of monolayer Al_2_CO and the Al_2_CO/Al_2_Se_3_ heterojunction. The calculations were carried out on pure materials and those containing vacancy defects in order to investigate the effects on structural, electronic, and optical properties for applications in photocatalysis.

### 3.1 Structural Properties

To study the effect of vacancies on Al_2_CO monolayer for application in photocatalysis, the structural parameters, including lattice constants, bond lengths, crystal structure, formation energy, and stability analysis, are calculated.^23^ The crystal structure of the Al_2_CO monolayer is hexagonal with space group P6m2. The unit cell, consisting of four atoms, has a volume of 42.9 Å^3^. The electronic configuration of Al in the outer shell is 3s^2^ 3p^1^ which shows an oxidation state of +3. For carbon, it is 2s^2^ 2p^2^ which symbolizes the +4 or −4 oxidation state.^24^ Oxygen has an electronic configuration as 1s^2^ 2s^2^ 2p^4^ soit forms a double bond with Al, and C makes a single bond with Al.

The structural relaxation of Al_2_CO unit cell leads to optimized lattice parameters: *a* = *b* = 3.48 Å, *c* = 3.02 Å, which are in good agreement with the reported values.^25^ In order to study the effects of vacancies and model the heterojunction, a 64-atom supercell (4 × 4 × 1) comprising 32 Al, 16 C, and 16 O atoms was prepared. The lattice constants of the monolayer are *a* = *b* = 12.88 Å and *c* = 11.15 Å. The calculated values of the bond lengths of Al–O and Al–C are 1.94 Å and 2.80 Å, respectively, with the thickness of the layer being 2.78 Å, which are in agreement with the corresponding reported values.^26^ The optimized structure of the monolayer of Al_2_CO is shown in Figure 1a–c. Al_2_Se_3_ as a 2D material with monoclinic structure in the *Cc* space group, is modeled. Al and Se atoms have respective oxidation states of +3 and −2. Al is observed to have two inequivalent sites in such a way that Al^3+^ bonds to four Se^2–^ atoms, forming tetrahedra via corner sharing. In the case of Se atoms, there are three inequivalent sites: the first is Se^2–^ which is bonded to two Al^3+^ atoms, forming a water-like geometry, whereas the other sites form three bonds to Al^3+^ giving a trigonal noncoplanar structure.

**Figure 1:**
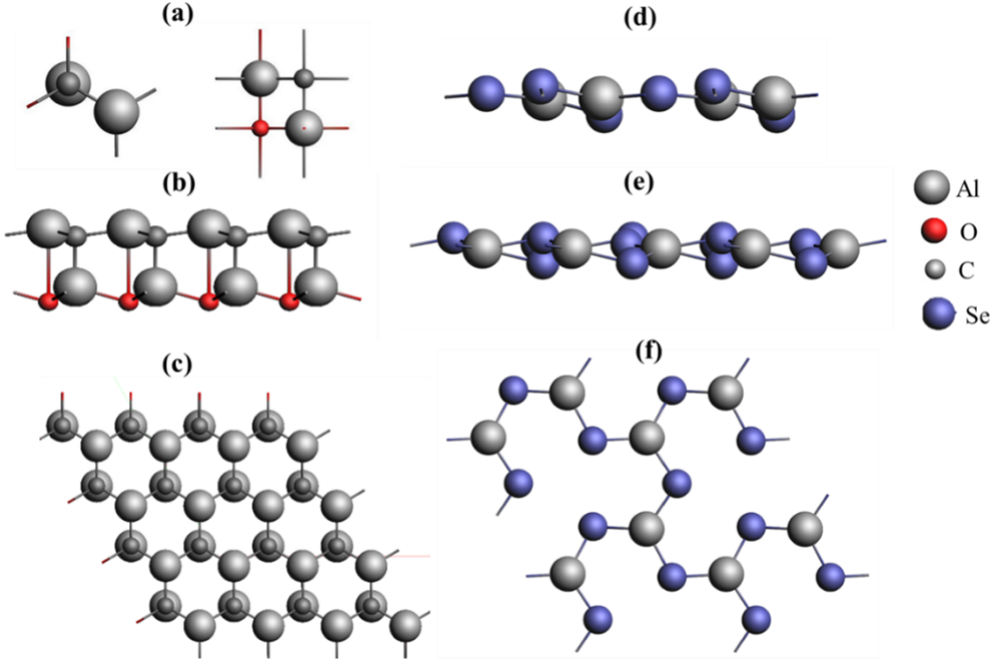
Crystal structure of Al_2_CO with (a) top and side views of the unit cell consisting of four atoms, (b) 4 × 4 supercell view along the *y*-axis, (c) 4 × 4 supercell view along the *z*-axis, (d) the crystal structure of the optimized 2 × 2 monolayer of Al_2_Se_3_ having a side view along the *x*-axis, (e) side view along the *y*-axis, and (f) top view along the *z*-axis.

The unit cell of bulk Al_2_Se_3_ consists of 5 atoms, with optimized lattice constants *a* = *b* = 6.71 Å and *c* = 5.82 Å. The unit cell is also optimized with periodicity as a slab, having an area of 39.1 Å^2^. For the purpose of the interface, the unit cell is transformed to form a supercell of 2 × 2, which gives a slab consisting of a total of 20 atoms, with 8 Al and 12 Se atoms. The optimized structure of the 2 × 2 Al_2_Se_3_ slab exhibited lattice constants of *a* = *b* = 13.42 Å and *c* = 11.64 Å, with an area of 156.3 Å^2^ as given in Figure 1d–f. The bond lengths between different atoms in the optimized structure are measured as Al–Al at 3.84 Å and Al–Se at 2.34 Å, whereas the dihedral bond angles Se–Al–Se and Al–Se–Al are found to be 119.2° and 113°, respectively.

The optimized structures of Al_2_CO and Al_2_Se_3_ were structurally joined at the interface and optimized to prepare a heterojunction in order to tailor the properties for photocatalytic applications.^27,28^ After optimizing both monolayers, the interface Al_2_CO/Al_2_Se_3_ is studied for photocatalytic activity. The heterojunctions are known to be successful when the lattice mismatch between the component materials is less than 5% at the interface.^29,30^ The lattice mismatch of heterojunction Al_2_CO/Al_2_Se_3_ found on the basis of the calculated lattice constants, appeared as 4.17%. The optimized structure of the resulting heterojunction, comprising 64 atoms in the Al_2_CO monolayer and 20 atoms in Al_2_Se_3_ is shown in Figure 2. The optimized interlayer distances Al–Al, Al–Se, and Al–C are calculated as 2.73, 3.64, and 2.05 Å, respectively, as shown in Figure 2e,f. The heterojunction, due to having an interlayer distance greater than 2.5 Å, is of the van der Waals type.^30^ The binding energy of the heterojunction (determined using eq 3) is found to be −5.349 eV.

3 $$E_{\mathrm{binding}}=E_{{Al}_{2}CO/{Al}_{2}{Se}_{3}}-E_{{Al}_{2}CO}-E_{{Al}_{2}{Se}_{3}}$$

**Figure 2:**
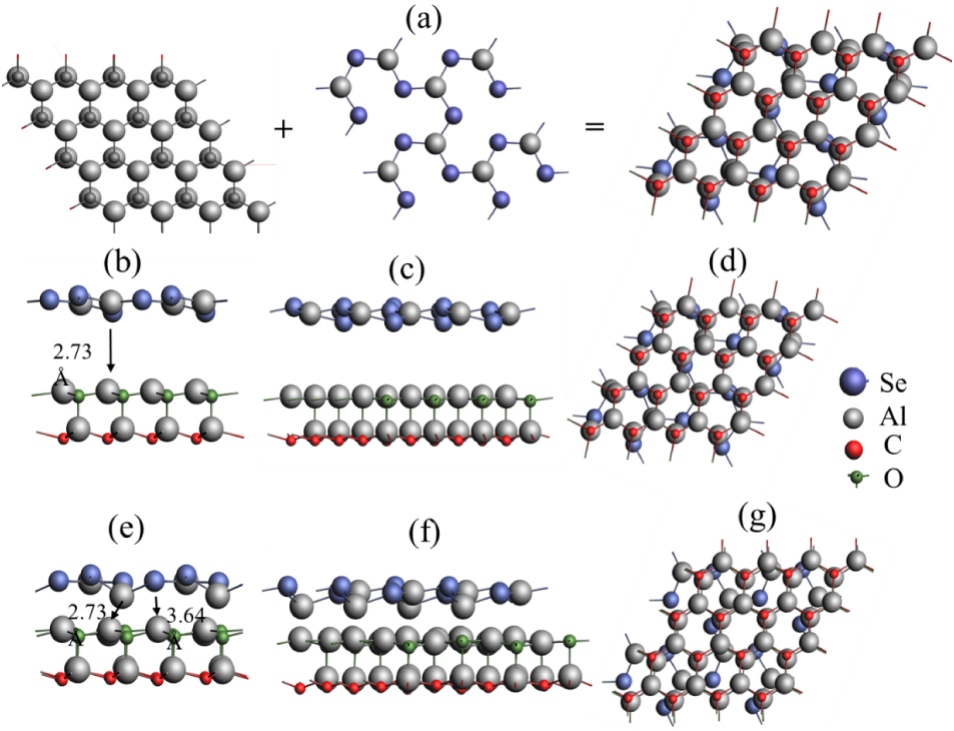
Structure of two monolayers forming the interface: (a) Al_2_CO/Al_2_Se_3_ (b) before optimization of the *x*-axis view, (c) the *y*-axis view, (d) the *z*-axis view, (e–g) the interlayer after optimization with interlayer distance ranging from 2.73 to 3.65 Å.

Where the terms indicate total energies of the optimized structures. The work of adhesion *W*_ads_ is the force that is required to separate a heterojunction into its component phases to a distance. *W*_ads_ may be negative or positive; if there is a higher and positive value of *W*_ads_ this means that the surfaces will bond more strongly. The value of *W*_ads_ is calculated using the formula given in eq 4 in order to determine the binding strength of the monolayers.

4 $$W_{\mathrm{adh}}=\frac{E_{1L}+E_{2L}-E_{\mathrm{int}}}{A}$$

Here, *E*_1L_ and *E*_*2*L_ are the total energies of the two respective layers that adhere together, *E*_int_ is the total energy of the optimized interface, and *A* is the area of the interface. The calculated value of *W*_adh_ in the case of the proposed heterojunction appeared to be 0.545 J/m^2^. The calculated value is slightly higher than that of the known van der Waals structures, having the values in the range of 0.2–0.5 J/m^2^.^31^ In order to further characterize the product, the interfacial energy is calculated via eq 5.

5 $$\gamma_{\mathrm{int}}=\sigma_{{Al}_{2}CO}+\sigma_{{Al}_{2}{Se}_{3}}-W_{adh}$$

where σ is surface energy (energy/area) and *W*_adh_ is the calculated work of adhesion. The value of *γ*_int_ is calculated as 3.243 J/m^2^ which points to the energy required to create the interface from bulk. The calculated value of interfacial energy is lower than several reported van der Waals heterojunctions, which is another indicator of the stability of the designed heterojunction.^32^ Al_2_CO is a two-dimensional layered material with distinct electrical and structural properties that set it apart from other metal oxides.^33^ Charge separation is encouraged by the type-II band alignment that forms in the Al_2_CO/Al_2_Se_3_ heterostructure, which is a desirable property that is not usually attained in conventional oxide systems. Despite having conceptual similarities to oxygen vacancies in TiO_2_, it is shown that the layered structure and covalent bonding environment of Al_2_CO produce different electrical changes.

#### 3.1.1 Intrinsic Defect Engineering

The material modification can be made in a number of ways, including control of morphology, selective usage of facet of crystal, engineering of defects, and formation of heterojunctions, etc.^34^ This study involves the investigation of intrinsic defects, Frenkel and Schottky vacancies, in the monolayer Al_2_CO and its interface with Al_2_Se_3_.^35^ From the literature, the favorable oxygen vacancy is formed by a reducing atmosphere or at low partial pressure of oxygen during synthesis, allowing the removal of O atoms readily without reoxidation. The vacancies that have been formed can be locked before healing by rapid cooling or quenching. Nanostructured materials having high surface area and hollow spheres tend to maximize surface oxygen vacancies. The sacrificial reagents play a part as chemical reductants, i.e., sodium borohydride, hydrazine, etc., and provide localized vacancy creation. Different characterization techniques help to detect the defect vibrations and unpaired electrons associated with the oxygen vacancies.^36^

The vacancy defects were created in the monolayer in order to determine their role in the properties of interest. In the case of the material Al_2_CO, we studied both the cation and anion vacancies on C, O, and Al sites. Further, the vacancy clusters in the form of the coexistence of both anion and cation vacancies, and Frenkel defects, were also investigated. The number of vacancy atoms in a material produced via any external agency can be estimated with the help of the formula. For our work, we performed the 0.01 intrinsic defect technique, i.e., 1/*n*, where *n* is the total number of atoms.^37^ The different types of intrinsic defects and their respective results are given in Table 1.

**Table 1:** Calculated Values of Structural Parameters, Formation Energy, and Band Gap Energy for the Al_2_CO Monolayer Having Different Types of Intrinsic Defects

Different defects in 4 × 4 × 1 monolayer of Al_2_CO	Bond lengths (Å)	Bond angles (Degree)	Formation energy *E_f_* (eV) ± 0.01 eV	Bandgap *E*_*g*_ (eV)
**Pure Monolayer**	Al–C: 1.939Al–O: 1.946	Al–O–Al: 111.9	–6.401	0.454
**O-vacancy**	Al–C: 1.934Al–O: 1.941	Al–O–Al: 111.7	–6.073	**0.855**
**2O-vacancy**	Al–C: 1.921Al–O: 1.940	Al–O–Al: 110.4	–6.259	0.904
**C-vacancy**	Al–C: 2.072Al–O: 2.795	Al–O–Al: 110.1	–7.028	0.695
**(O and C)-vacancy**	Al–C: 3.154Al–O: 3.821	Al–O–Al: 108.4	–6.286	**1.043**
**Frenkel defect “Al”**	Al–C: 2.012Al–O: 2.211	Al–O–Al: 109.2	–6.404	0.792
**Al-vacancy**	Al–C: 1.946Al–O: 1.913	Al–O–Al: 112.1	–6.358	0.000

The comparison revealed that the electronic bandgap increased (except for Al vacancies) in the cases of intrinsic vacancy defects. In the case of the Al cationic vacancy, the bandgap decreased to zero, which indicates a semiconducting-to-metallic transition of the material. The analysis of bond lengths and angles, along with the formation energy for the Al vacancy, indicated that the structural stability of the material is not disturbed. The observed change in electronic character is favorable for many other applications, such as hydrogen storage and utilization as an electrode in metal-ion batteries. The Frenkel defect is created by displacing the Al(30) atom from its site and placing it between Al(50)–C(63)–Al(64). In the optimized structure, Al appeared to attach with the carbon sites C(47)–C(31).

The anionic vacancy on the O and C sites appeared to increase the band gap. For the case of the O-vacancy, no notable changes in bond lengths are observed, which points to the structural stability of the material. The oxygen divacancy was also tested, but the calculated formation energy and structural parameters point toward its unsuitability. The carbon vacancy also led to favorable results, which included more active sites and regulation of the band structure. The coexistence of both the O and C vacancies may improve charge separation by the formation of a built-in electric field, but the stability of the material is questioned as per our computational details. Among all the defects studied herein, the oxygen monovacancy is found to be energetically suitable to proceed further with calculations on photocatalytic activity. Surface oxygen vacancies are known to enhance photogenerated carrier separation and offer more localized electrons to the absorbed water.^38^ In the next sections, attention will be given to discuss the calculated stability and band edge alignment in the case of the material with favorable anionic vacancies. For the intrinsic anion vacancies of O and C sites, the optimized lattice parameters are the same, with slight changes in bond lengths as given in Table 1. The structure of the Al_2_CO monolayer with O and C vacancies is given in Figure 3a.b, respectively.

**Figure 3:**
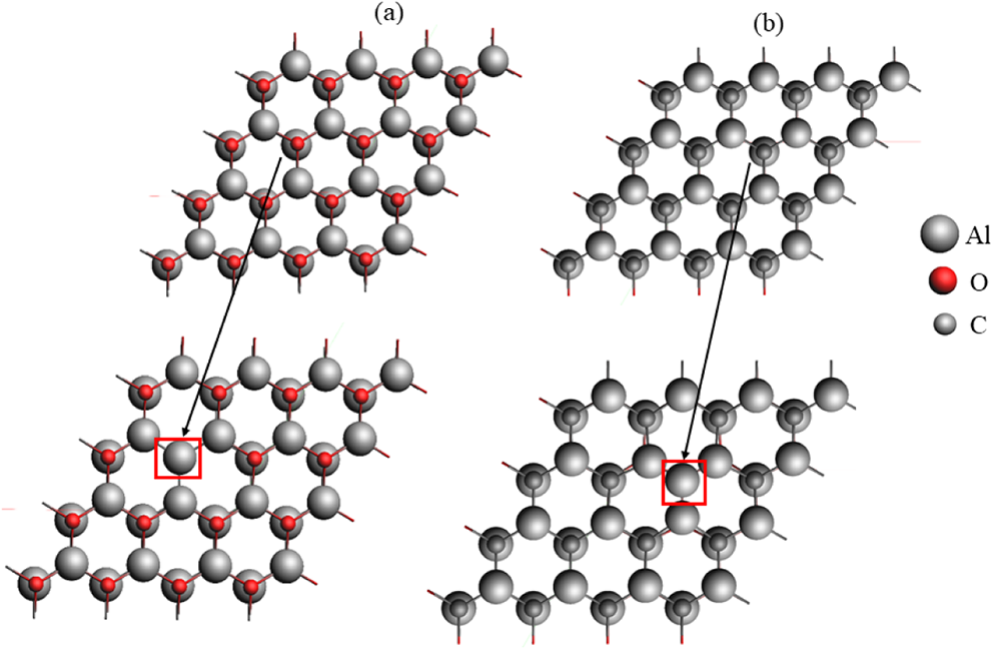
Defect-containing monolayer Al_2_CO: (a) oxygen vacancy and (b) carbon vacancy highlighted in a box.

#### 3.1.2 Stability Analysis of Al_2_CO and Heterojunction

The stability of a material is important to confirm its suitability for various applications. In this regard, the binding energy of Al_2_CO is calculated by using the formula given in eq 6.

6 $$E_{b}=E_{Al_{2}\mathrm{CO}}-\left(nE_{Al}+nE_{C}+nE_{O}\right)$$

where *E*_Al2CO_ is the total energy of the monolayer Al_2_CO; *E*_Al_, *E*_C_, and *E*_O_ are the energy values corresponding to Al, O, and C atoms, while *n* is the number of respective atoms in the monolayer. The average binding energy for the monolayer is calculated as −0.954 eV, which points to its stability. The negative value of the energy indicates exothermic conditions, which points to monolayer’s stability. In order to check the dynamical stability of the monolayer, the phonon dispersion curves were also calculated and displayed in Figure 4. The entire modes in the phonon spectrum calculated for the Al_2_CO monolayer are positive, which confirms its dynamic stability. The phonon modes are computed via the response function, which indicates perturbation in the vibration. The number of vibrational modes can be determined by the 3*n* degrees of freedom, with *n* being the number of total atoms in the structure. Dynamic stability refers to the restoration of atomic positions to their original state under the application/removal of external forces. These findings point to the structural and dynamical stability of the monolayer, on the basis of which it is safe to proceed to further investigations. The vacancies may introduce gap-trapping states, which can interact with adsorbed water molecules. The vacancy creates an active site, and hence symmetry is changed. The stability of the defective monolayer can be studied by the calculation of formation energy and phonon spectra. The calculated defect formation energy of O-vac Al_2_CO is −6.073 eV, whereas that of C-vac Al_2_CO is −7.028 eV. The negative value of this energy confirms the exothermic conditions and thus the thermodynamic stability. The dynamical stability after the formation of carbon and oxygen vacancies is investigated using the phonon spectra, as given in Figure S1a,b, respectively. The phonon dispersion curves for the O-vac case, as given in Figure S1a, clearly indicate the absence of any negative mode. On the other hand, the phonon spectrum calculated for the C-vac in the Al_2_CO monolayer, as given in Figure S1b, display minor negative modes of frequency. Hence, the C-vacancy appears to exhibit less dynamic stability in comparison to that of the O-vac in the Al_2_CO monolayer. On the basis of dynamic stability calculated via phonon spectra, we proceed to further investigations by using the O-vac-based Al_2_CO monolayer.

**Figure 4:**
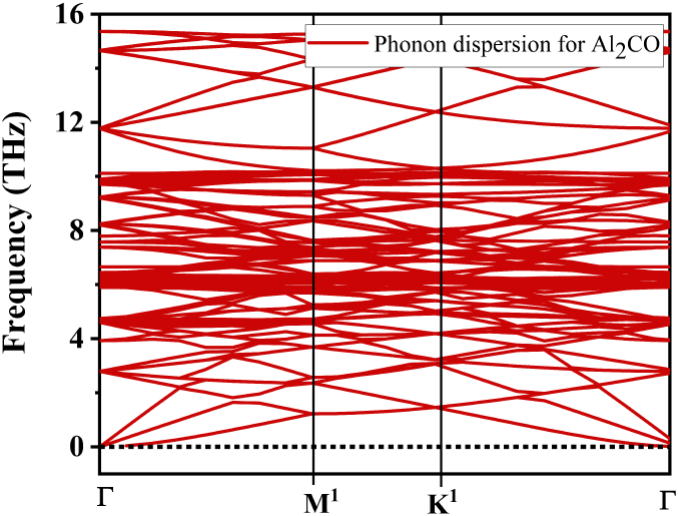
DFT-calculated phonon dispersion curves for the Al_2_CO monolayer.

The stability analysis of heterojunction is very important when considering its applications into account.^38^ In this regard, the calculations of phonon dispersion curves and ab initio molecular dynamics (AIMD) simulations were carried out to investigate the respective dynamical and thermal stabilities of the heterojunction Al_2_CO/Al_2_Se_3_. The phonon dispersion curves shown in Figure 5a indicate the absence of any imaginary modes, which confirms the dynamic stability of the heterojunction Al_2_CO/Al_2_Se_3_.

**Figure 5:**
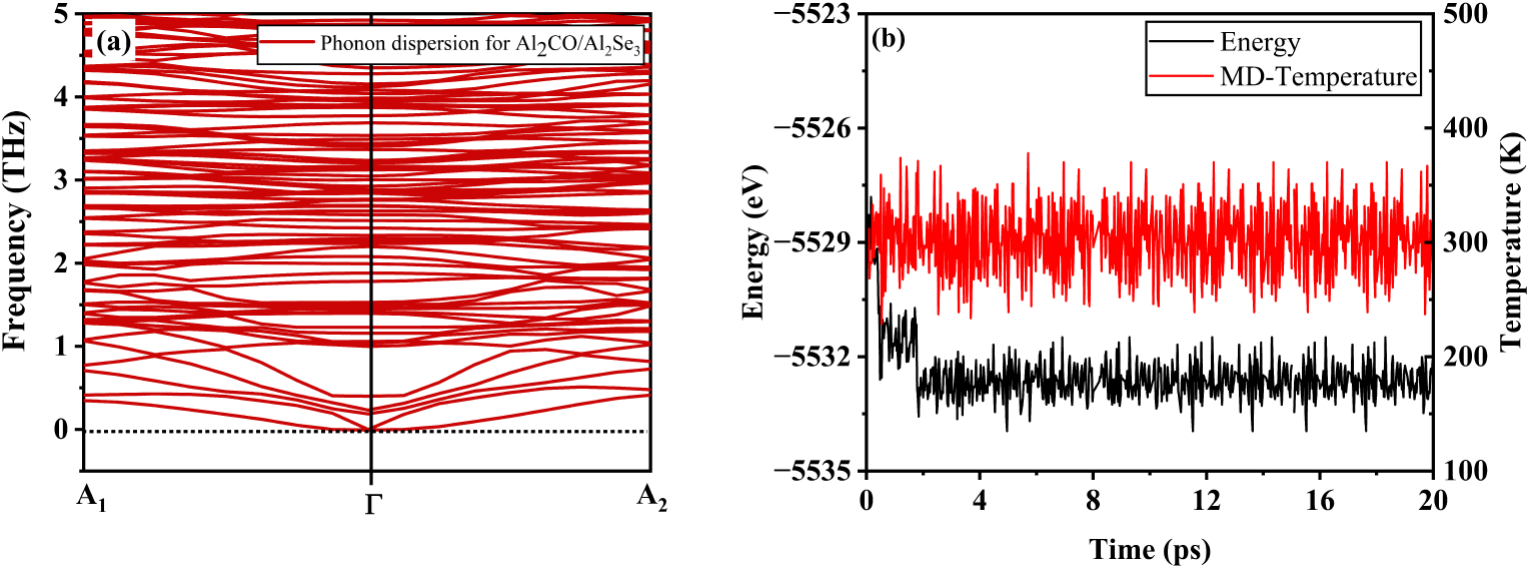
(a) DFT-calculated phonon dispersion curve representing the dynamical stability of interface Al_2_CO/Al_2_Se_3_. (b) The molecular dynamics of the interface Al_2_CO/Al_2_Se_3_ at room temperature (300 K).

The molecular dynamics (MD) process minimizes energy to evaluate the thermal properties of materials at a finite temperature.^39^ The heterojunction Al_2_CO/Al_2_Se_3_ was exposed to room temperature, 300 K, using the thermostat to see the respective effects on the structure as well as energy fluctuations. The AIMD results calculated for the heterojunction are represented in the form of energy and temperature versus the time step, as given in Figure 5b. The graph clearly shows that the temperature and energy oscillate around a mean value. There is no abrupt change in the temperature, as the fluctuations are very small in comparison to the MD temperature. Similarly, the energy curves, after reaching the equilibration phase, continue to oscillate in a very small energy window. These oscillations were observed for 20 ps, and the corresponding structure remained intact, as no bond breakage or structural degradation was observed. These findings point to the dynamic and thermal stability of the heterojunction, which demonstrates the likelihood of its effective utilization in photocatalysis.

### 3.2 Electronic Properties

The band structure calculations provide useful information to characterize electronic materials.^40^ The electronic properties of the individual monolayers and the heterojunction were calculated in order to investigate the material’s properties for photocatalytic applications. The electronic bandgap of the Al_2_CO calculated at the GGA-PBE-D3 level of theory, is 0.45 eV, as indicated in the band diagram given in Figure 6a. The valence band maxima (VBM) and conduction band minima (CBM) lie at the same symmetry point of the Brillouin zone, which points to the direct bandgap of the material.^41^ After the creation of a vacancy, the band structure is greatly modified, as the bandgap is increased to 0.85 and 0.69 eV for oxygen and carbon vacancies, respectively, as shown in the band diagrams given in Figure 6b,c, respectively. The CBM and VBM for O-vac in Al_2_CO lie on the S and Γ symmetry points, respectively, giving rise to an indirect bandgap. This increase in the bandgap is useful for regulating the electronic properties and hence utilizing the material in photocatalytic applications. On the other hand, the band structure of the C-vac in Al_2_CO shows a direct bandgap of only 0.69 eV. Hence, the O-vac in Al_2_CO appears suitable for modulating the electronic properties of the monolayer from an application point of view. The band structure calculated for the supercell of Al_2_Se_3_ is shown in Figure 6d, which points to a direct bandgap of 2.92 eV that agrees with the literature.^33^

**Figure 6:**
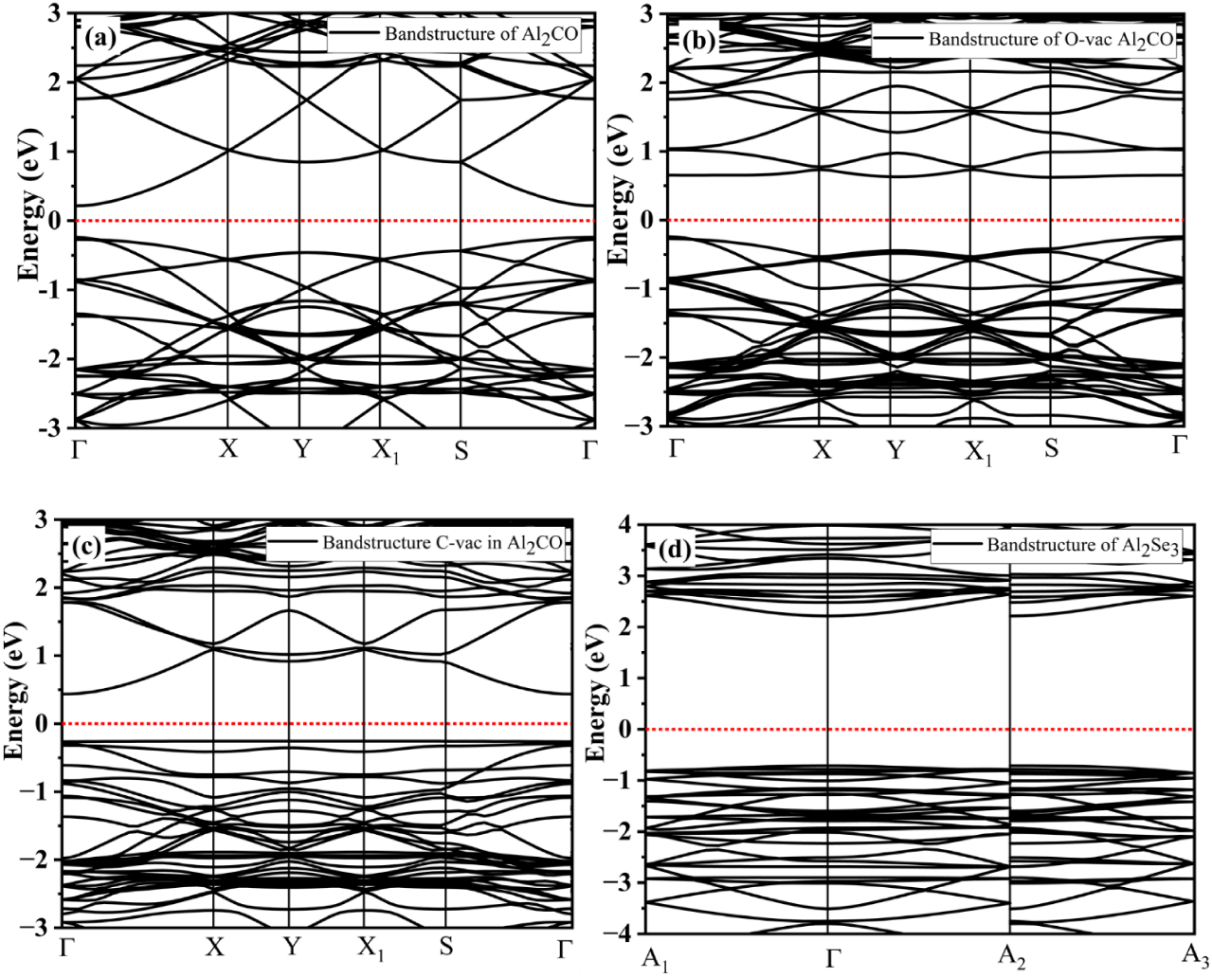
DFT-calculated electronic band structure diagram of (a) Al_2_CO monolayer, (b) oxygen vacancy in Al_2_CO monolayer, (c) carbon vacancy in Al_2_CO, (d) and the 2 × 2 monolayer of Al_2_Se_3._ The high-symmetry points are shown on the *x*-axis and energy in eV is shown on the *y*-axis, where the Fermi level is shifted to 0 eV.

The total and partial DOS of the perfect and vacancy-containing monolayers is analyzed in order to have a deeper understanding of the electronic characteristics. The states related to O and C mostly occupy the Al_2_CO valence band, whereas Al states primarily occupy the conduction band as given in Figure S1.1a. The effect of creating vacancy on the band structure is clear from the PDOS. In Figure S1.1b,c, the change in the Al and C contributions after the creation of the O vacancy maximizes the contribution of C-p around the Fermi level. In the case of a C vacancy in the Al_2_CO monolayer, the C states in the valence band are reduced. In the O-vac, small DOS are found near Fermi level in the conduction band, but for the C-vac, this region becomes populated. The observed changes play a role in changing the band gap and band type, which leads to favorable results when photocatalysis is taken into account. Hence, the electronic properties also corroborate with the structural properties and stability analysis. according to which the oxygen vacancy in Al_2_CO appears suitable to exploit the material for photocatalytic activity. Taking these considerations into account, the interface Al_2_CO/Al_2_Se_3_ is investigated in detail for photocatalytic water splitting and the hydrogen evolution reaction (HER).

The electronic band structure of the material provides necessary information on energy levels, forbidden gap, conduction band minima, and valence band maxima against high-symmetry points of the Brillouin zone. The band structure of Al_2_CO/Al_2_Se_3_ is shown in Figure 7a, which depicts its semiconducting character with a direct bandgap of 0.754 eV as the CBM and VBM lie on the Γ point. The interface exhibits dominant p states, whereas the s character is determined in the conduction band away from the Fermi level. This electronic structure was observed to change after the introduction of an intrinsic defect in the heterojunction. On the basis of the previously mentioned stability and energetics, the introduction of an oxygen vacancy in Al_2_CO part of the heterojunction will be studied.

**Figure 7:**
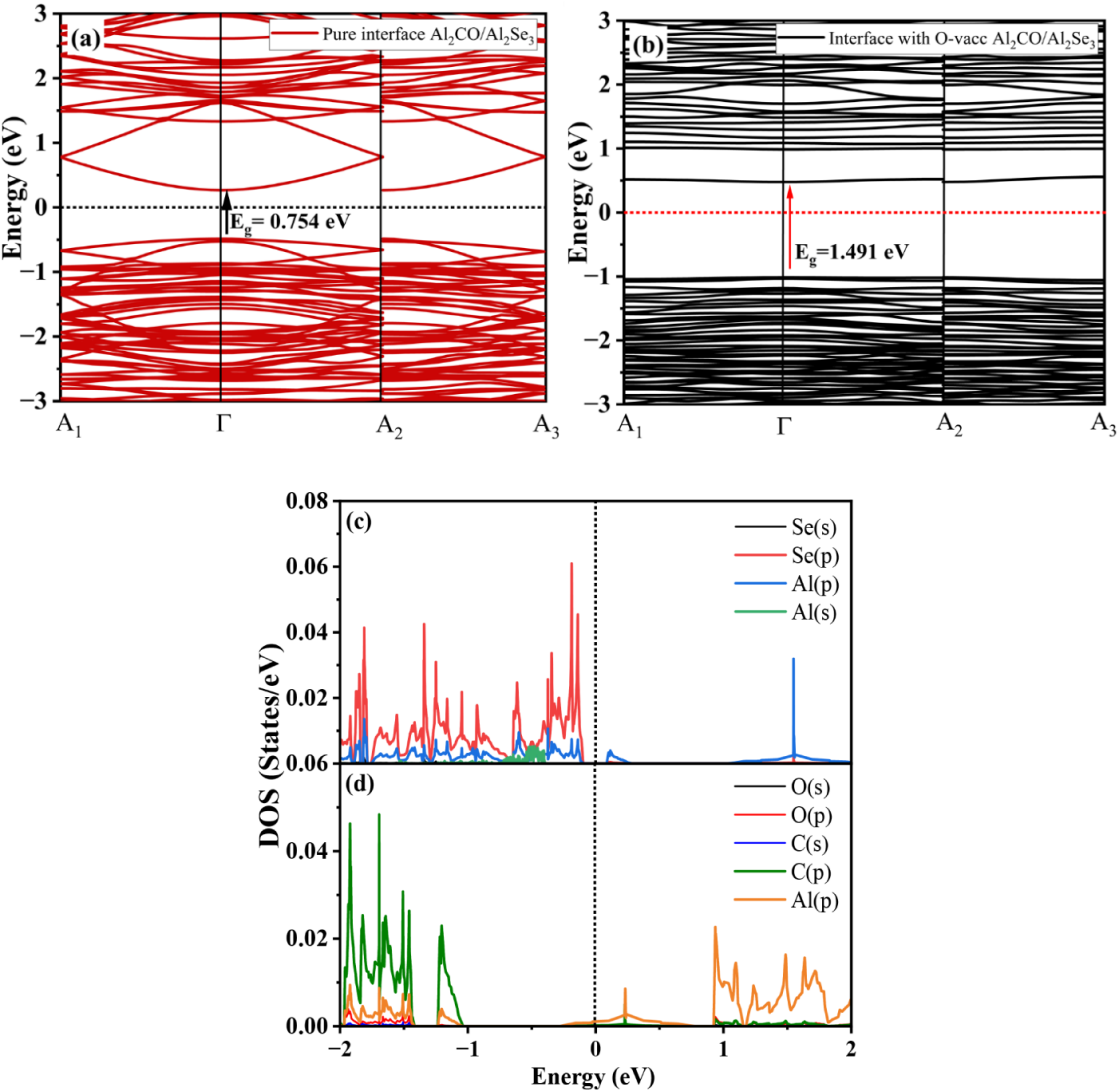
Band structure calculated for (a) heterojunction Al_2_CO/Al_2_Se_3_ with a direct bandgap of 0.754 eV, (b) heterojunction Al_2_CO/Al_2_Se_3_ after the introduction of a vacancy, (c) the partial DOS of Al_2_Se_3_ monolayer with major contributions from the p orbital of Se (d), and Al2CO monolayer with major contributions from the p orbital of C.

The suitability of the oxygen vacancy containing heterojunction is analyzed in light of calculated DOS. The total DOS calculated for the interface before and after introduction of oxygen vacancies is shown in Figure S.1.2. The VB of the heterojunction is mainly occupied by the states related to Al_2_CO monolayer, with other interfacial contributions. It can be seen that no states are present on or near the Fermi level. The PDOS calculated for the Al_2_Se_3_ monolayer is given in Figure 7c. The VB comprises the Se-p states, with the dominance starting after the Fermi level, in agreement with literature.^42^ The dominant contribution of Al-p states in the CB is observed with a populated area found after 2 eV. The PDOS of Al_2_CO shows the major contribution of C-p states in the VB and Al-p states in the CB. Hence, the electronic structure of the heterojunction reveals a major contribution of Se-p from Al_2_Se_3_ and C-p from Al_2_CO. The electronic properties of the vacancy-containing heterojunction are analyzed in the following.

The introduction of an oxygen vacancy in the Al_2_CO part of the heterojunction appeared to increase the band gap to 1.491 eV, as represented in Figure 7b. The vacancy broadened the forbidden gap below the Fermi level as the VBM shifted downward, which enhances the separation of photogenerated charge carriers. The energy level of the VBM, which was at −0.5 eV, shifts to −1.0 eV. This effect has been observed for several semiconducting materials, whereas increase in the band gap tends to inhibit the carrier recombination.^43,44^ The resulting band gap of the heterojunction lies in the range ∼1.23–3.2 eV, suitable for photocatalytic water splitting.^45^ The total DOS of the O-vac containing heterojunction, given in Figure S1.3d–f, clearly shows that the contribution of carbon is shifted near the Fermi level in the VB.^46^ The vacancy regulation of the electronic properties thus plays an important role in enhancing the photocatalytic activity of the heterojunction.

### 3.3 Charge Analysis

The mechanism of charge transfer in materials can be investigated via electron localization and Hirshfeld (HF) charge analysis. The net amount of charge on Al, C, and O atoms is calculated with the help of electron density from the surface to the isolated atom, as shown in Figure 8. The HF values for the pure, O-vac Al_2_CO, and the heterojunction Al_2_CO/Al_2_Se_3_ are given in Figure 8a–c, respectively. Al atoms, being highly positive, transfer electronic charge to C and O atoms, which have a negative charge afterward. The positive value of HF charge points to the ability of charge transfer to lower charge values. Al atoms have the same uniform positive charge of about 0.473 e, as represented by the blue color, but after the creation of the O-vac, as shown in Figure 8, it can be seen that the surrounding Al atoms have a positive value of about 0.238 e. The other Al atoms tend to have an increased positive value of 0.477 e. Hence, the charge transfer is affected by the creation of the vacancy and plays an important role in the electronic character of the material. The charge transfer character inside the heterojunction was also studied by an HF charge analysis. The charge distribution observed at the Al_2_CO/Al_2_Se_3_ interface, where Al atoms exhibit the highest positive charge (+0.479 e) and Se atoms show a negative charge (−0.102  e), is consistent with previous theoretical and experimental studies on III–VI and III–IV compounds. Similar trends in charge transfer have been observed, where aluminum typically acts as an electron donor due to its electropositive nature, while chalcogen atoms like Se act as electron acceptors. The significant charge polarization at the interface, as visualized in Figure 8c, supports the formation of a built-in electric field, which has been discussed as a crucial factor in tuning the electronic properties in heterostructures. Hence, Al_2_CO happens to accept the charge from Al_2_Se_3_ to electronically stabilize the heterojunction after charge transfer. The magnitude of the charge difference between the HF values of the most positive and negative one is 0.009 e, which suggests that the interface has vdW interactions, in agreement with the structural properties.

**Figure 8:**
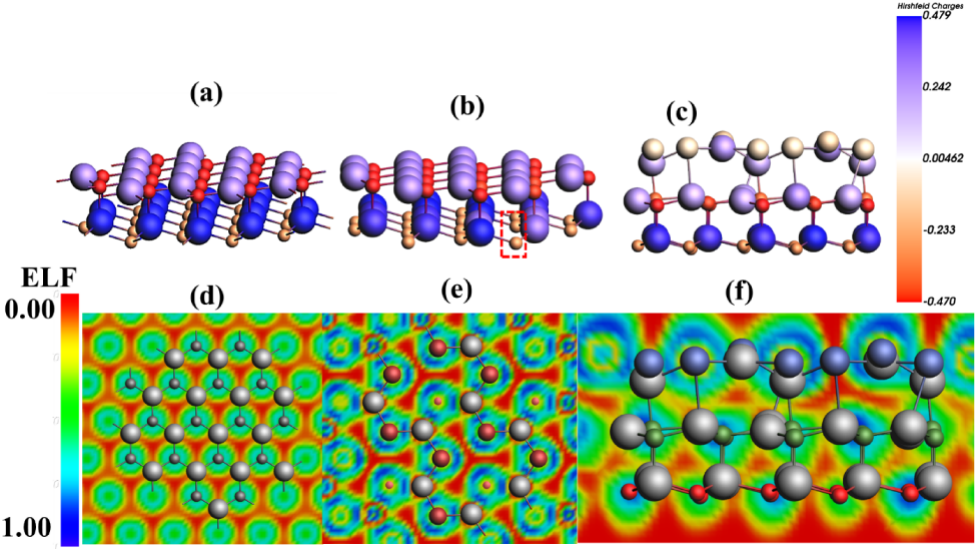
Hirshfeld charge analysis of (a) Al_2_CO monolayer, (b) O-vac-based Al_2_CO monolayer, (c) Al_2_CO/Al_2_Se_3_ with the highest positive value represented by blue color and the lowest by red. The electron localization function with a scale bar from 0 to 1 is represented by (d) top view of Al_2_CO monolayer, (e) top view of Al_2_Se_3_, and (f) side view of the interface Al_2_CO/Al_2_Se_3_.

The bonding character in the materials is further studied with the help of the electron-localization function (ELF), which provides a colored graphical representation of electron density for different regions. It is a resourceful tool to study how electrons delocalize in solids to shed light on the bonding scenario.^47^ When the result is renormalized to a value between 0 and 1, the ELF shows the structure of the atomic shells. ELF = 0 denotes very low charge density, indicating absence as in metallic bonds, while ELF = 1 represents perfectly localized electrons, as in covalent bonds. The value ELF = 0.5 denotes entirely intermediate delocalized electrons.^48^ Figure 8d–f represents the ELF analysis of Al_2_CO, Al_2_Se_3_, and the Al_2_CO/Al_2_Se_3_ interface, respectively. The localized areas should be identified to explain the density contributions. There are two regions found in the Al_2_CO monolayer, i.e., around the C and O atoms and another near the Al atoms. The electron density near Al is zero due to its electropositive nature. The near-zero electron density around Al atoms is consistent with their well-known electropositive character, as observed in other Al-based compounds such as Al_2_O_3_ and AlN, where Al donates electron density to more electronegative elements. In contrast, in Al_2_Se_3_, the relatively high electron density near Se atoms (ranging from 1.00 to 0.75 au) indicates significant electron localization. This observation aligns with previous studies that have reported strong anionic character and electron accumulation around Se due to its higher electronegativity and larger atomic radius. Such charge localization is often associated with ionic–covalent bonding behavior in III–VI semiconductors.^24^ Furthermore, the bonding is also predicted with the help of electronegativity χ as per the formula given in eq 7.

7 $$\chi ={[\chi {\left(A\right)}^{p}\chi {\left(B\right)}^{q}\chi {\left(C\right)}^{r}]}^{1/p+q+r}$$

where *A*, *B*, and *C* represent different atoms in a compound, and *p*, *q*, and *r* indicate total number of atoms present in the compound. The electronegativity for both layers is calculated, and the difference between them is used to predict and explain the bonding character. The value of χ for Al_2_CO is 4.696, and the χ for Al_2_Se_3_ is 4.620, which indicates an electronegativity difference of 0.076. The small electronegativity difference points to a nonpolar bond and hence a vdW type heterojunction.

### 3.4 Band Edge Alignment

The photogenerated carriers appearing after the irradiation of light onto the surface of a semiconductor play a primary role in photocatalytic activity. The suitability of the photocatalyst is basically checked via the position of the band edges in the band structure of the material. The band edge alignment of a photocatalyst should match the redox potential for photocatalytic activities. In order to check the suitability for water splitting, we calculated the normal hydrogen electrode (NHE) potential representing the band edge positions.^49^ The VBM and CBM, to be represented on the NHE scale, were calculated using the formulas given in eqs 8 and 9.

8 $$E_{\mathrm{CBM}}=\chi -E_{e}-0.5E_{g}$$

9 $$E_{\mathrm{VBM}}=E_{g}+E_{\mathrm{CBM}}$$

where χ is the absolute electronegativity, *E_g_* is the bandgap, and *E_e_* is the energy of the free electron. The value of electronegativity is calculated using eq 7.

Figure 9 shows the band edge alignment of the Al_2_CO monolayer and its respective vacancy-based heterostructure for the hydrogen evolution reaction (HER). The potential of HER is −4.44 eV for the photocatalyst, and it must be satisfied for the reduction of water.^50^ The oxygen vacancy in the monolayer greatly influences the electronic properties and band alignments. The Al_2_CO slab does not satisfy the reduction potential, but for the O-vac Al_2_CO, the CB is more negative than the reduction potential of 4.44 eV. Hence, the potential is satisfied, and the photo-generated charge carriers will migrate to H_2_O to reduce it into H^+^ and hence H_2_.^51^ The band alignment computed for the interface indicates that the redox potential is satisfied. The CBM for the interface before and after vacancy is 4.09 eV and −4.27 eV , respectively, which is more negative than the reduction potential. The VBM for O-vac-based Al_2_CO/Al_2_Se_3_ is −5.75 eV, whereas the heterojunction without vacany does not satisfy the oxidation potential as it has the value 5.00 eV. The pH-dependent alignment can be determined using the formulas *E*_CBM_= −4.44 + pH× 0.059 eV and *E*_VBM_ = −5.67 + pH× 0.059 eV.^52^ The redox potential, including the pH, can be represented using these formulas from 0 to 7 pH.^53^ Hence, the photocatalytic activity can be initiated to migrate the charge carriers to assist the water-splitting mechanism for the O-vac Al_2_CO/Al_2_Se_3_ which is further explained in the following sections.

**Figure 9:**
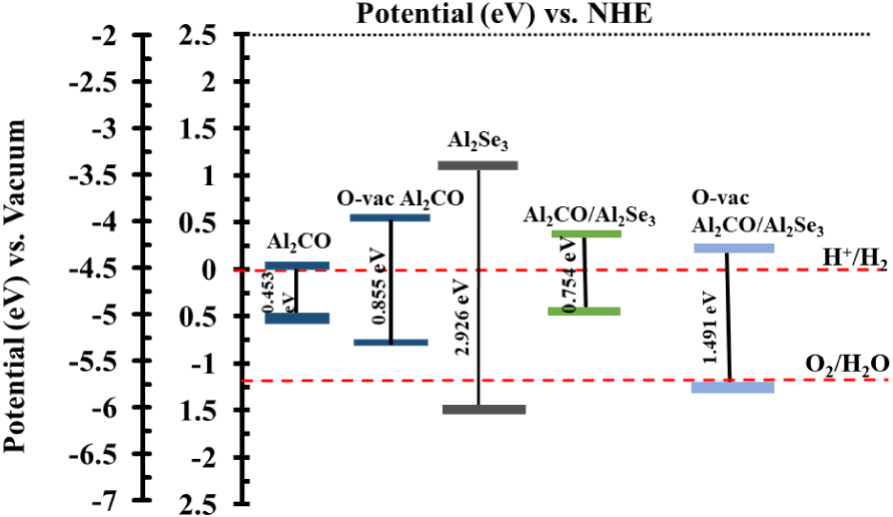
Band edge alignment for HER in the case of Al_2_CO monolayer before and after the introduction of the O vacancy and the heterojunction Al_2_CO/Al_2_Se_3_ before and after the introduction of the O vacancy.

### 3.5 Photocatalytic Water Splitting

The process of photocatalysis involves illuminating a photocatalyst with photons coming from the sun or an artificial source, which assist to drive the redox reactions of interest.^54^ The vacancy-based Al_2_CO and interface Al_2_CO/Al_2_Se_3_ are studied for usage in photocatalytic water splitting. The incident photons produce electron–hole pairs by shifting the electrons from the VB to the CB of the semiconducting photocatalyst.^55,56^ Free electrons (e^–^) and holes (h^+^) are generated in the CB and VB, respectively. These photogenerated charge carriers play their roles in water’s oxidation and reduction processes. The site consisting of an excess of electrons acts as a hydrogen evolution site, whereas the one consisting of holes acts as an oxygen evolution site.^57^ Furthermore, the reaction pathway for the water molecule adsorbed to the surface of the heterojunction was studied by using the Nudged Elastic Band (NEB) method. The minimum energy pathway of ionic diffusion in a solid and in a certain chemical reaction can be identified by using it. The different transition states for the reaction pathway can be investigated via optimization on the interface.^58^ The identification of an imaginary frequency mode in the vibrational spectrum confirms the presence of a transition state, a widely accepted criterion in computational chemistry for verifying that a reaction path has passed through a saddle point on the potential energy surface. This indicates that the transformation of water into a hydroxyl (OH^–^) and a proton (H^+^) has successfully overcome the activation barrier. The calculated reaction energy of 9.71 kJ/mol (∼0.1 eV) suggests a thermodynamically favorable step, consistent with previous DFT studies investigating elementary steps in the HER on similar catalyst surfaces. Similar reactions on Ni-based catalysts show barriers in the range of 0.35–0.55 eV. Such energy barriers have been reported for efficient HER catalysts, highlighting the low overpotential required for proton reduction.^59^ These findings contribute to understanding the underlying mechanism of HER, which will be discussed further in the context of both hydrogen and oxygen evolution reactions in the following section.

#### 3.5.1 Redox Reaction

The process of water splitting occurs as a redox reaction, where oxidation and reduction simultaneously take place. The photocatalytic activity of the catalyst for the HER is modeled on the basis of Gibbs energy (Δ*G*). The steps involved in the HER are given in eq 10.

$$*+\left(H^{+}+e^{-}\right)\Rightarrow H^{*}$$

10 $$H^{*}+\left(H^{+}+e^{-}\right)\Rightarrow H_{2}+*$$

The H^+^ ion, after the absorption of a photogenerated electron, converts into intermediate H*, and this again undergoes the first process to form H_2_ molecules. The HER, predicted via the graph of Gibbs free energy, can be calculated by the formula given in eq 11.

11 $$\Delta G=\Delta E_{H}+\Delta E_{\mathrm{ZPE}}-T\Delta S$$

where Δ*S* represents the change in thermodynamic entropy at a standard temperature of 298.15 K; Δ*E*_ZPE_ denotes the change in zero-point energy, and Δ*E_H_* corresponds to the change in total energy for the system involving the interface with absorbed hydrogen, the pristine interface, and the isolated H_2_ molecule, as referenced in^51^. These parameters are critical in evaluating the free energy change (Δ*G*) associated with hydrogen adsorption, a key step in the hydrogen evolution reaction (HER). The calculated Δ*G* values for two distinct adsorption sites—namely, the regions near aluminum (Al) and selenium (Se) atoms—are illustrated in Figure 11a. A positive value of Δ*G* indicates that the adsorption process is thermodynamically unfavorable under standard conditions, implying that the reaction will only proceed when an external energy source is applied to overcome the energetic barrier, as explained in ref. 52. In contrast, negative or near-zero Δ*G* values suggest that the hydrogen adsorption process is either spontaneous or requires minimal energy input, thus being thermodynamically feasible. This trend aligns with the Sabatier principle, which posits that for optimal catalytic performance, the interaction between the surface and adsorbates (such as H^∗^) should be neither too weak nor too strong.^60^

Specifically, the Δ*G* values calculated for the Se and Al sites are −0.144 eV and −0.091 eV, respectively, demonstrating that hydrogen adsorption is energetically more favorable on the Se site. The more negative ΔG value for the Se site suggests stronger binding and a more exothermic reaction pathway, enhancing the likelihood of HER occurring at this site. For comparison, perfect catalysts like Pt (0 eV) are known to have an optimal free energy for hydrogen adsorption (Δ*G*_H^∗^) near zero, but 2D materials like MoS_2_ and g-C_3_N_4_ have Δ*G*_H^∗^ values of roughly 0.1–0.2 eV and 0.6–0.8 eV, respectively. Comparable HER activity is indicated by the calculated Δ*G*_H^∗^ for the Al_2_CO/Al_2_Se_3_ heterostructure (∼0.09–0.14 eV), which falls in the theoretical range for active MoS_2_ edge sites. Furthermore, the effect of applying an external electrochemical potential is also examined. At an applied potential of *U_e_* = 1.23 eV, the corresponding Δ*G* value is represented by a blue line for the Se site in Figure 11a. This scenario simulates realistic operating conditions during HER, where an external potential is used to drive the reaction. The results show that even under such conditions, the HER remains favorable, particularly at the Se site, reinforcing its potential as an active catalytic site. Overall, the thermodynamic data suggest that the Se site exhibits superior activity for HER due to its more negative Δ*G*, indicating a more exothermic and thus energetically favorable reaction profile.

Furthermore, the splitting of water molecules into O and OH by the mechanism of a redox reaction is shown in Figure 11b, where state number 5 of the potential energy surface (PES) symbolizes the favorable redox reaction. The PES signifies the surface energy as a multidimensional graph representing the collection of molecules with respect to the function of their position.^60^ The single water molecule and the cluster of molecules follow the reaction pathway in the same manner, which indicates the reactivity and possible transformations of the molecules, as shown in Figure 11b.^61^

The OER pathway on the catalyst surface has been systematically investigated, revealing that the OER process is kinetically more challenging than the HER. The OER proceeds via a four-step mechanism involving sequential proton-coupled electron transfer (PCET) events. The elementary reaction steps can be represented as follows:

12 $$*+H_{2}O\to HO^{*}+H^{+}+e^{-}$$

13 $$HO^{*}\to O^{*}+H^{+}+e^{-}$$

14 $$O^{*}+H_{2}O\to HOO^{*}+H^{+}+e^{-}$$

15 $$HOO^{*}\to O_{2}+H^{+}+e^{-}$$

The asterisk sign ^∗^ indicates the site for intermediates over the surface of the photocatalyst to absorb, as schematically represented in Figure 10. The Gibbs free energy is determined for every step, and the graph is plotted, as given in Figure 11c. This indicates that the free energy changes for the four elementary steps of the OER, denoted as Δ*G*_1_, Δ*G*_2_, Δ*G*_3_, and Δ*G*_4_, are 1.06, 0.96, 0.41, and 2.31 eV, respectively. These values suggest that all the individual reaction steps involved in the OER process are exothermic under the given conditions, which is a favorable indication for catalytic activity. Among the reaction intermediates, the hydroxyl species (OH^∗^) exhibits the most negative adsorption energy of −1.27 eV, indicating strong binding to the catalyst surface. This comparatively high adsorption strength implies that OH^∗^ is more strongly adsorbed than other intermediates, such as O^∗^ and OOH^∗^, whose adsorption energies are calculated to be −0.63 eV and −0.36 eV, respectively. The strong adsorption of OH^∗^ facilitates one of the four steps in the OER pathway, making it energetically favorable. However, while this step benefits from favorable energetics, it is important to consider that the overall OER efficiency depends on the complete free energy landscape encompassing all four reaction steps.

**Figure 10:**
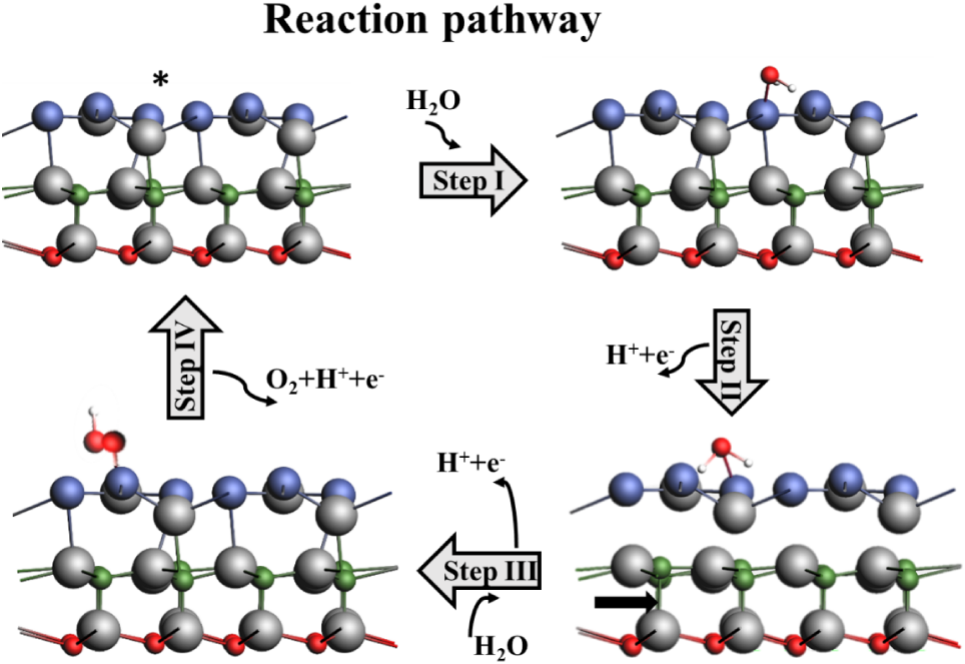
The reaction pathway of the adsorbed H_2_O molecule on the interface is represented in the diagrams involving four steps.

**Figure 11:**
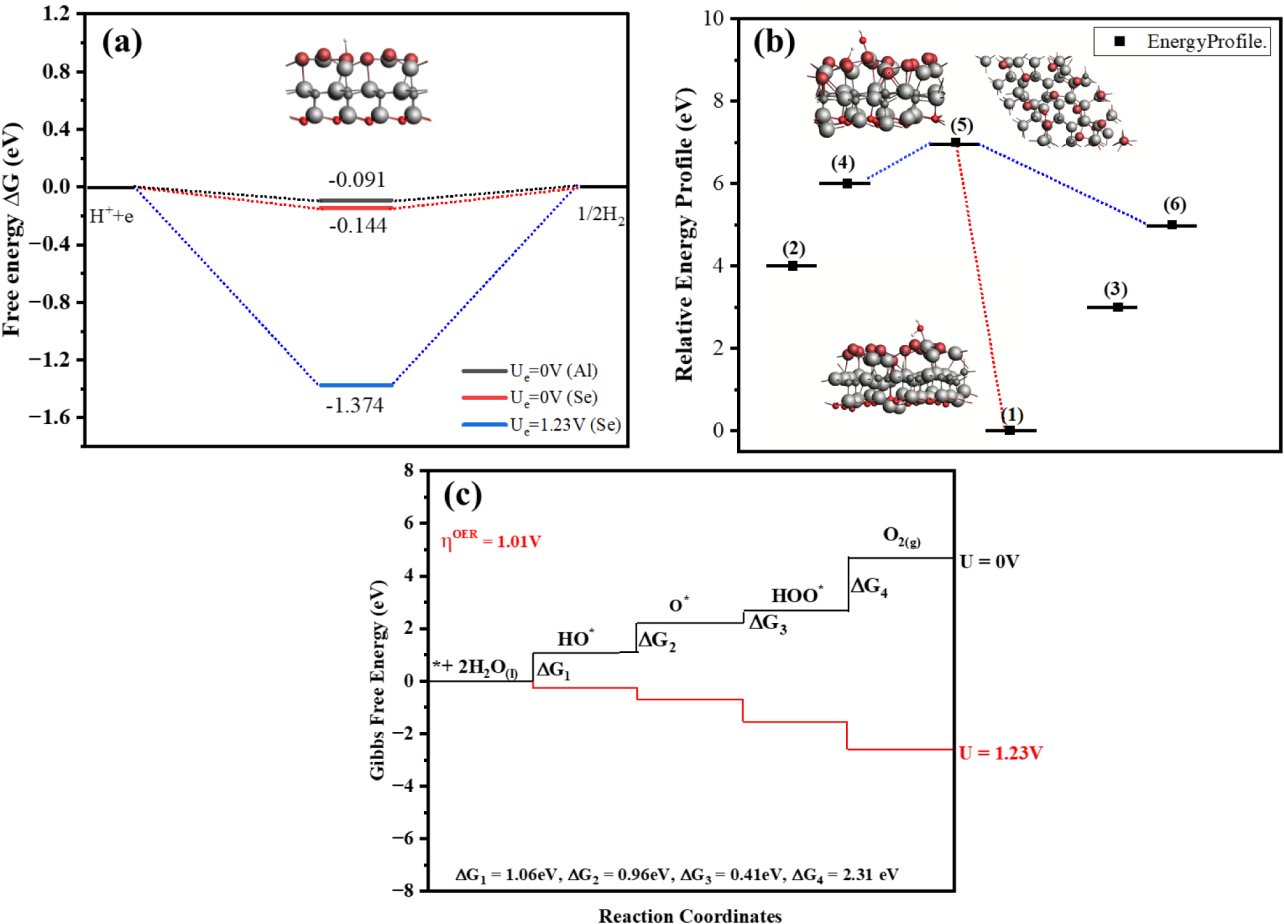
(a) HER representation for two different sites, Al and Se, on the interface Al_2_CO/Al_2_Se_3_. (b) The energy states representing the evolution of oxygen from water on the surface of O-vac Al_2_CO/Al_2_Se_3_ into O and OH at state 5. (c) Gibbs free energy diagram for the OER elementary steps at zero potential and at external potential 1.23 eV.

Despite three steps showing moderate Δ*G* values, the fourth step, which corresponds to the transformation of the HOO^∗^ intermediate into molecular oxygen (O_2_), emerges as the most energy-demanding step with the highest Δ*G* of 2.31 eV. This step becomes the potential-determining step (PDS) of the OER process, as it introduces the largest thermodynamic barrier among the four. Figure 11c graphically illustrates this energy profile, highlighting the energy-intensive nature of the final step. The high Δ*G* value for this transformation implies that a significant driving force or applied potential is required to complete the reaction, even though the reaction is still exothermic.

The theoretical overpotential is the excess potential needed to overcome the energy barrier or PDS compared to the thermodynamic equilibrium potential of 1.23 V. It also reflects the cumulative effect of all reaction steps, including the energy losses due to charge transfer resistance. The highest value of Δ*G* (i.e., Δ*G*_max_) along the reaction pathway is used to find this theoretical minimum overpotential. The value of the theoretical minimum overpotential (i.e., $\eta_{\mathrm{OER}}^{\mathrm{DFT}}$ ) can be determined via the calculated value of the PDS Δ*G*_max_ per eq 16:

16 $$\eta_{\mathrm{OER}}^{\mathrm{DFT}}=\frac{\Delta G_{\max}}{e}-1.23V$$

The calculated value of PDS of 2.31 eV leads to a theoretical minimum overpotential of 1.08 V for the O-vac containing Al_2_CO/Al_2_Se_3_ interface. Specifically, overpotentials for similar catalysts are typically reported in the range of ≈0.75–0.98 V^62^ and for photocatalytic water oxidation on oxynitrides/oxides, theoretically, such as the work on NaTaO_3_ vs SrTaO_2_N shows predicted overpotentials of ∼1.30 V (oxide) vs ∼1.01 V (oxynitride).^63^ The value is compared to several reported OER catalysts, which points to a balance of intermediate absorption energies, although the final step represents the kinetic bottleneck of the reaction pathway. The calculated low overpotential indicates the proposed material for use as a stable electrocatalyst for water oxidation.

### 3.6 Optical Properties

The optical properties are important when the photocatalytic performance of materials is taken into account. The catalyst should absorb the major portion of sunlight from the visible to infrared parts of the spectrum.^64^ The absorption depends on the dielectric function, and the value of the absorption coefficient α­(ω) is calculated using eq 4. The absorption spectra calculated for the monolayers and the O-vac heterojunction are shown in Figure 12. The O-vac Al_2_CO/Al_2_Se_3_ appeared to offer absorption peaks from UV to IR spectra. The visible light harvesting is of interest in practical photocatalysis applications.^65^ It can be seen that Al_2_Se_3_ exhibits strong absorption only in the ultraviolet region (λ < 430 nm), consistent with its wide bandgap (∼2.92 eV). In contrast, Al_2_CO displays absorption extending into the near-infrared region due to its narrow bandgap (∼0.45 eV), but such low-energy transitions are insufficient for water splitting. The Al_2_CO/Al_2_Se_3_ heterojunction shows a significant red shift of the absorption edge into the visible range (λ ≈ 450–750 nm). The analysis of the calculated absorption spectra points to activity in the visible region. It shows that the heterojunction exhibits a value of ∼1.30 × 10^5^ cm^–1^ near the ∼2.50 eV energy, which is stronger when compared with both the monolayers.^51^ Hence, the heterojunction Al_2_CO/Al_2_Se_3_ is capable of absorbing a higher intensity of light in the visible to infrared region of the electromagnetic spectrum.

**Figure 12:**
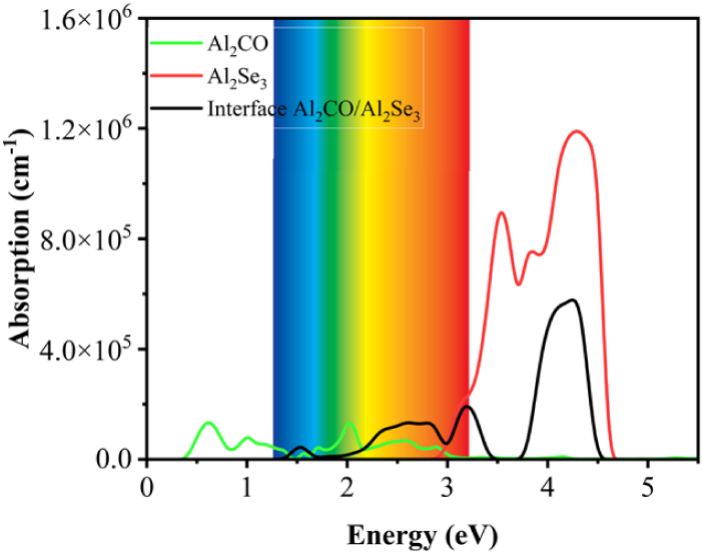
Calculated absorption spectra for monolayers and heterostructure Al_2_CO/Al_2_Se_3_.

### 3.7 Modeling Defect Concentration under Synthesis Conditions

Based on the theoretical findings of this work, we are able to predict the probable O-vacancy concentration from a thermodynamic perspective using the calculated defect formation energy and the comparison of different defect types, as given in Table 1. The calculated value of defect formation energy corresponding to the single O-vacancy is found as −6.073 eV (under the most stable conditions, which are O-poor). This highly negative value indicates that the creation of the oxygen vacancy in the material is spontaneous under a highly exothermic process. This observation points to the presence of a strong thermodynamic driving force to produce a high concentration of O-vacancies during the synthesis of the material or thermal annealing in an oxygen deficient atmosphere. The favorable O-vacancy formed by a reducing atmosphere or low partial pressure of oxygen during the synthesis allows the removal of the O atoms readily without reoxidation. The vacancies that have been formed can be locked before healing by rapid cooling or quenching. The nanostructured materials, due to having a high surface area, tend to maximize the surface oxygen vacancies. The sacrificial reagents can act as chemical reductants, i.e., sodium borohydride, hydrazine, etc., to provide localized vacancies.

The primary OER analysis in this study focused on modeling a single isolated O-vacancy, which represents a low-to-moderate concentration regime in the material. Such a concentration is considered optimal for catalysis because it ensures the boosting of the active isolated sites without structural degradation caused by the vacancies and related lattice strain occurring in the mentioned regime. The structural changes triggered by O-vacancies induced changes in bond lengths and angles, as given in Table 1. Further, our findings indicate that the O-vacancy caused a notable increase in bandgap from 0.454 to 0.855 eV when compared with similar single C and Al vacancies in the material. This significant change in electronic structure, based on charge transfer, further justifies the modeling of low-concentration vacancies to tailor the catalytic properties of the material.

Al_2_CO is a 2D layered material with distinct electrical and structural properties that set it apart from other metal oxides. The charge separation is encouraged by the type-II band alignment that forms in the Al_2_CO/Al_2_Se_3_ heterostructure, which is a desirable property that is not usually attained in conventional oxide systems. Despite having conceptual similarities to oxygen vacancies in TiO_2_, it is shown that the layered structure and covalent bonding environment of Al_2_CO produce different electrical changes. This study demonstrates a 2D van der Waals heterojunction formed by two distinct materials, Al_2_CO and Al_2_Se_3_, which points to advancements over the reported metal oxide monolayers and heterojunctions, with salient features as: (i) the interface Al_2_CO/Al_2_Se_3_ and the engineering of its physical properties based on the introduction of defects to exploit OER catalysis. (ii) The modeling of O-vacancy in the Al_2_CO layer, where the synergistic electronic effects from the other layer Al_2_Se_3_ provide unique active sites for catalysis when compared to single-component bulk materials. (iii) For shedding light on the distinction of the studied heterostructure, a quantitative comparison is given to make an experimental follow-up of the presented theoretical results. (iv) The monolayers studied herein exhibited lower overpotential than several conventional monolayer catalysts, giving values like η_OER_ *=* 1.67 V (MoSSe), η_OER_ *=* 2.99 V (MoS_2_), and η_OER_ = 1.34 V (IrO_2_). Similarly, the overpotential calculated for our heterostructure is 1.08 V, which is smaller than several reported heterostructures like η_OER_ = 1.85 V (MoSSe/P-doped graphene), η = 1.48 V (WS_2_/MoS_2_), and η = 1.18 V (BN/Carbon).^58,59^ Though experimental values are often lower, this low theoretical overpotential suggests a highly favorable intrinsic catalytic activity of the heterostructure. (v) The findings are not restricted to the introduction of the defect but also explain the mechanism referring to the superior performance, which is usually missing in DFT studies on contemporary oxides. (vi) The proposed interface offers a near-ideal Gibbs free energy that minimizes the energy differences between key intermediates.

## 4 Summary

In summary, this work reports theoretical investigations on the prospects of intrinsic vacancy defects in 2D Al_2_CO and its interface Al_2_CO/Al_2_Se_3_. The findings indicate that the anion vacancies (O, C) are favorable for the proposed heterojunction on the basis of calculated electronic structure, charge analysis, and transport properties. Among O and C vacancies, the O-vac Al_2_CO exhibited a bandgap increased to 0.885 eV, whereas the interface O-vac Al_2_CO/Al_2_Se_3_ offered a direct bandgap of 1.491 eV. The rate of photo-generated charge carriers increases for the vacancy-based material. The band edge alignments of the O-vac Al_2_CO/Al_2_Se_3_ satisfy the hydrogen evolution potential, which points to its suitability for photocatalytic water splitting. The activity of HER, calculated by the Gibbs free energy (Δ*G*), is −0.144 eV, which symbolizes that the reaction is spontaneous and exothermic. Furthermore, the optical absorption coefficient forming the absorption spectra indicates that the interface of the O-vac Al_2_CO/Al_2_Se_3_ exhibited good absorption in the visible region, which is greater than that of the individual monolayers. These findings of the study point out that oxygen vacancies in Al_2_CO and the heterojunction Al_2_CO/Al_2_Se_3_ produce a favorable environment for enhanced photocatalytic water splitting. However, these are the findings under idealized conditions based on DFT, and the stability and synthetization of vacancy-based Al_2_CO/Al_2_Se_3_ heterostructure require further investigation. Future work should focus on the experimental validation of defect formation and its interface fabrication, along with more advanced simulations.

## Supplementary Material



## Notes

The DFT-calculated phonon spectra for (a) C vacancy in Al_2_CO monolayer and (b) O vacancy in Al_2_CO monolayer. The calculated total and partial DOS, with the Fermi level shifted to 0 eV for (a) the pure 4 × 4 × 1 Al_2_CO monolayer, (b) the oxygen vacancy in Al_2_CO, (c) carbon vacancy in Al_2_CO. Electronic properties: (a) the partial DOS of Al_2_Se_3_ monolayer, with a major contribution from the p orbital of Se. (b) Al_2_CO monolayer with a major contribution from the p orbital of C. (c) The total DOS of the interface (d) and (e) show the electronic contribution of atoms in the monolayers Al_2_Se_3_ and Al_2_CO respectively (PDF)
