# Supplementary material for: Defect-Engineered Al2CO/Al2Se3 Heterostructure for Enhanced Photocatalytic Water Splitting
Source: ACS Omega. 2025 Dec 23;11(1):1447–60. doi: 10.1021/acsomega.5c09075 (PMC12809572; doi:10.1021/acsomega.5c09075)
Supplement: Supplementary file 1 [file ao5c09075_si_001.pdf]

Defect-Engineered Al_2CO/Al_2Se_3 Heterostructure for Enhanced Photocatalytic Water Splitting

Iram Shahzadi[1], Abdul Majid[*,1], Bisma Wasim[1], Mohammad Alkhedher[2], Ahmed Ahmed Ibrahim[3], Sajjad Haider[4], Kamran Alam[*,5]

[1]Department of Physics, University of Gujrat, Gujrat 50700, Pakistan

[2]Mechanical and Industrial Engineering Department, Abu Dhabi University, Abu Dhabi 59911, United Arab Emirates

[3] Department of Physics and Astronomy, College of Science, King Saud University, P.O. Box 2455, 11451 Riyadh, Saudi Arabia

[4]Chemical Engineering Department, College of Engineering, King Saud University, P. O. Box 800, Riyadh 11421, Saudi Arabia

[5]Department of Chemical Engineering Materials Environment Sapienza university of Rome, Italy

*Correspondence: Abdulmajid40@yahoo.com ; Kamran.alam@uniroma1.it

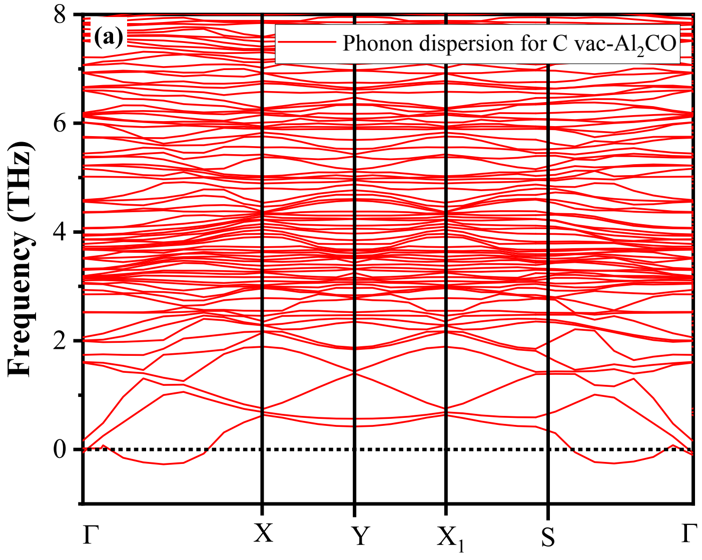

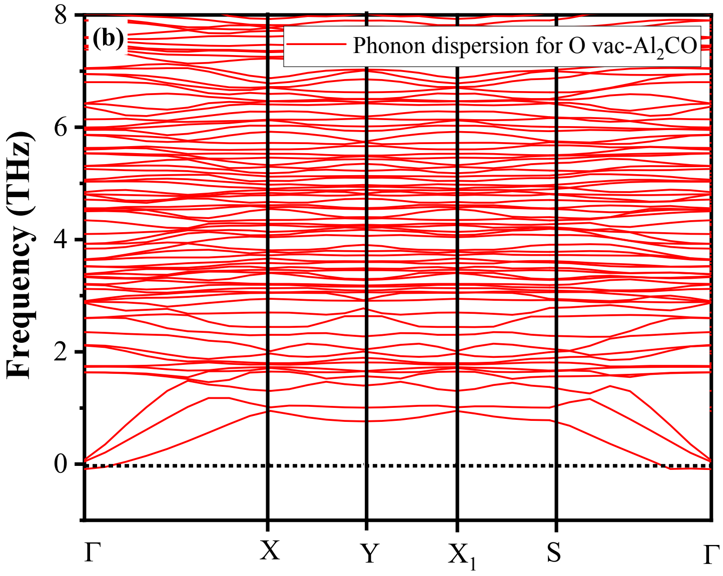

Figure S.1.1: The DFT calculated phonon spectra for (a) C vacancy in Al_2CO monolayer (b) O vacancy in Al_2CO monolayer.

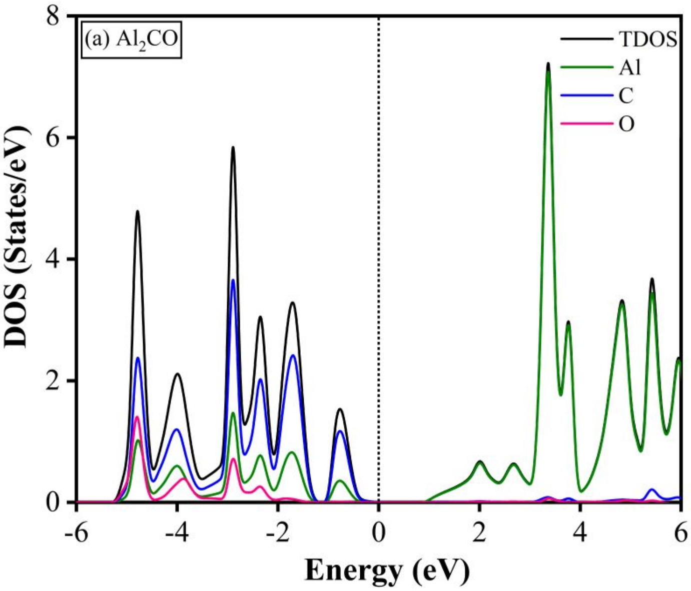
(a) Al_2CO
TDOS
Al
C
O
DOS (States/eV)
Energy (eV)

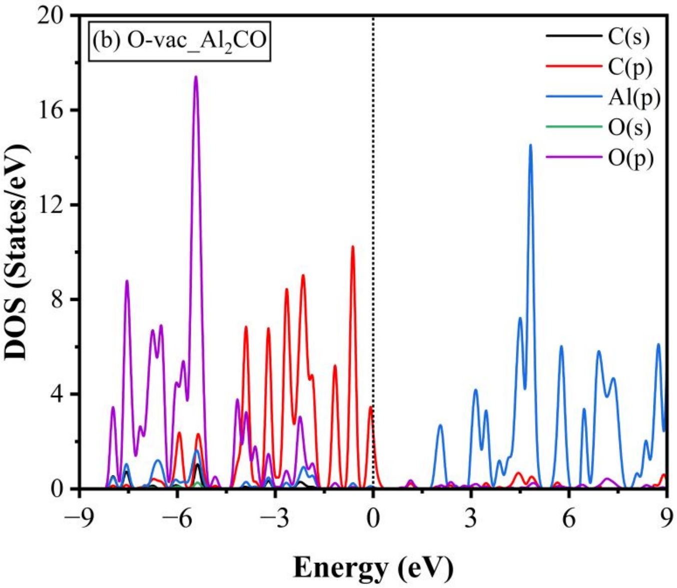
(b) O-vac_Al_2CO
C(s)
C(p)
Al(p)
O(s)
O(p)
DOS (States/eV)
Energy (eV)

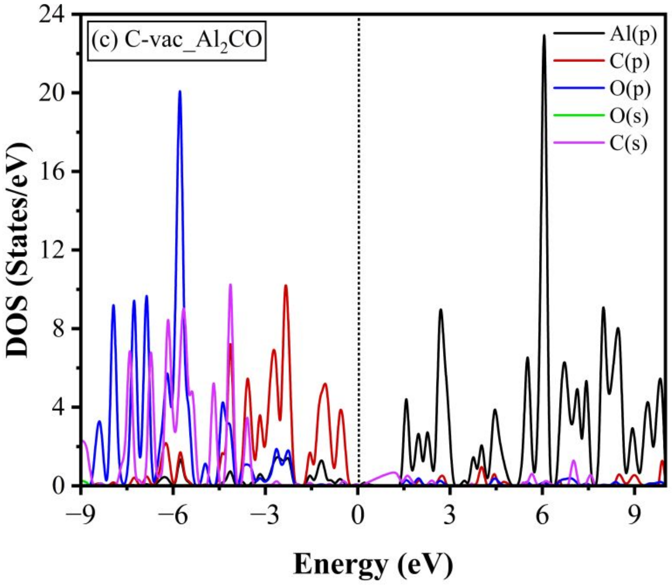

Figure S.1.2: The calculated total and partial DOS with Fermi level shifted to 0 eV for (a) The pure 4×4×1 Al_2CO monolayer (b) Oxygen vacancy in Al_2CO (c) Carbon vacancy in Al_2CO.

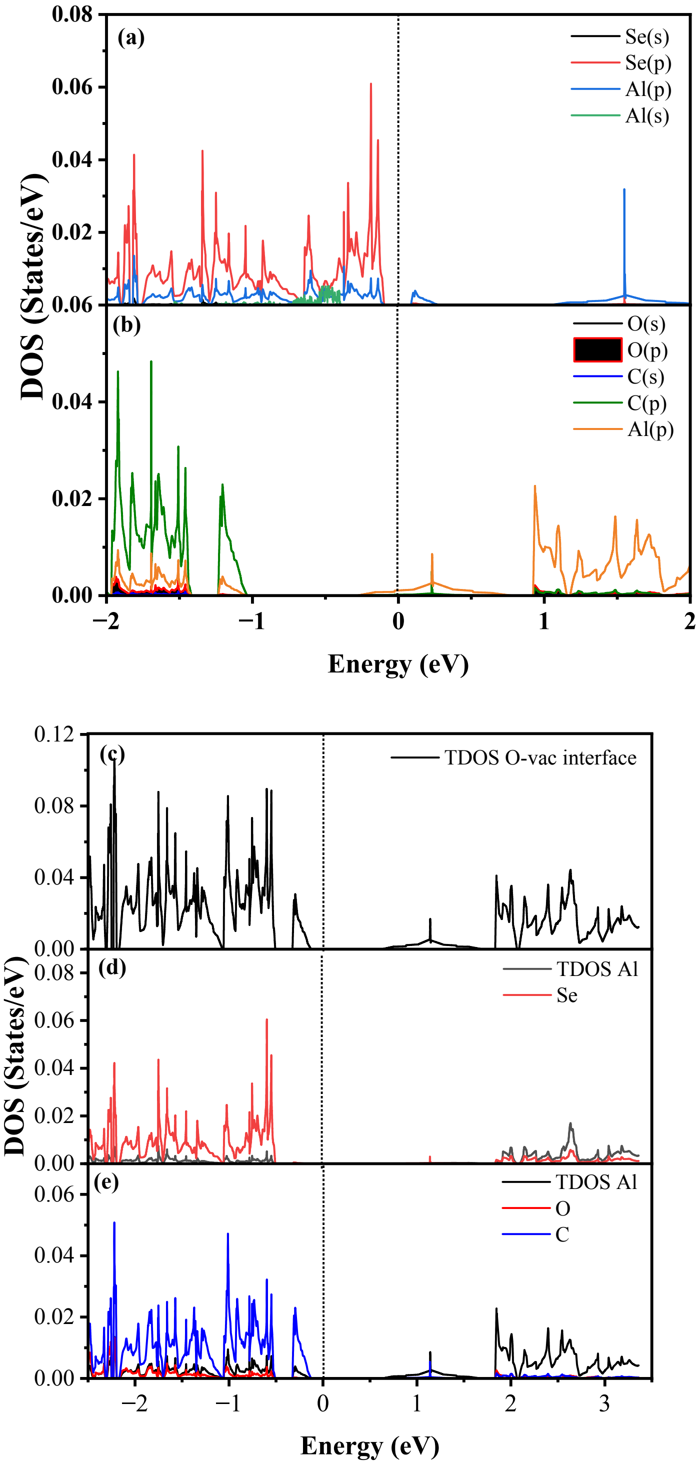

Figure S.1.3: Electronic Properties (a) The partial DOS of Al_2Se_3 monolayer with major contribution of p orbital of Se (b) Al_2CO monolayer with major contribution of p orbital of C, (c) total DOS of interface (d) and (e) shows the electronic contribution of atoms in the monolayers Al_2Se_3 and Al_2CO respectively.
